# Items

**Published:** 1908-02

**Authors:** 


### ITEMS.

The State Civil Service Commission will hold examinations February 15, 1908; for among others the following positions: Chemist, Department of Agriculture, $1,200.00; Keeper, Erie County Penitentiary or Jail, $780.00 to $840.00; Orderly, Erie County Hospital, $540.00 and maintenance; Physician, State

Hospitals, $900.00 and maintenace; Trained Nurse, State Institutions, $420.00 to $600.00 and maintenance.

The last day for filing applications for these examinations is February 8th. Full information and application forms may be obtained by addressing Charles S. Fowler, Chief Examiner, Albany.

---

Examination for Physician (Male) Panama Canal.—The United States Civil Service Commission announces an examination on February 19-20, 1908, at Buffalo, to secure eligibles from which to make certification to fill vacancies as they may occur in the position of physician, at $150.00 per month, in the Panama Canal Service. It is probable that about fifteen appointments will be made, this estimate being based upon the number of appointments made during the past year.

The examination will consist of the subjects mentioned below, weighted as indicated: (1) Letter-writing, 5; (2) anatomy, 5; (3) therapeutics, 5; (4) physical diagnosis (including questions relating to tropical diseases), 25; 5 general pathology and practice (including questions relating to tropical diseases), 25; (6) bacteriology and hygiene, 5; (7) obstetrics and gynecology, 5; (8) practical experience, 25. Total, 100.

Applicants must indicate in their applications that they are citizens of the United States, graduates of recognised medical schools, and have had at least one year's experience as interne in a general hospital. Persons lacking the above qualifications will not be admitted to the examination.

The element of experience will be rated upon the statements made in application, Form 1312, and special credit will be given to physicians who show that they have been for more than a year members of the house staff of large metropolitan hospitals. Men only will be admitted to this examination. Age limit, 20 to 45 years on the date of the examination.

Applicants should at once apply either to the United States Civil Service Commission, Washington, D. C., or to the secretary of the board of examiners at any place mentioned in the list printed hereon, for application Form 1312. The medical certificate in form 1312 must be filled in by a reputable practising physician other than the applicant. No application will be accepted unless properly executed and filed with the Commission at Washington prior to the hour of closing business on February 10, 1908. In applying for this examination the exact title as given at the head of this announcement should be used in the application.

Issued January 13, 1908.

